# Cholecystopleural fistula: A case report and literature review

**DOI:** 10.1097/MD.0000000000039366

**Published:** 2024-08-16

**Authors:** Yong Yang, Qian Chen, Yi Hu, Liangsong Zhao, Pengcheng Cai, Suqi Guo

**Affiliations:** Department of General Surgery, The First People’s Hospital of Shuangliu District, Chengdu/West China Airport Hospital of Sichuan University, China.

**Keywords:** case report, ERCP, fistula, gallbladder pleural fistula, gallstone, pleural fistula

## Abstract

**Introduction:**

Gallstone with acute cholecystitis is one of the most common diseases in the clinic. If the disease is serious, gallbladder gangrene, perforation, and sepsis may be caused. Gallbladder diseases rarely cause thoracic-related complications, especially pleural fistula, which is very rare in clinical practice.

**Patient concerns:**

A 52-year-old male patient was admitted to the emergency department for 1 month with recurrent right middle and upper abdominal pain.

**Diagnosis:**

Computed tomography diagnosis: cholecystitis and peri-inflammation, small abscess around the base of the gallbladder, local peritonitis, and bilateral pleural effusion.

**Interventions:**

After admission, conservative treatment was given. On the 4th day of admission, the symptoms worsened, and an emergency catheter drainage was performed on the right thoracic cavity to extract 900 mL of dark yellow effusion. After the operation, a large amount of bili-like fluid was continuously drained from the thoracic drainage tube. After the iatrogenic biliary fistula caused by thoracic puncture was excluded, cholecystopleural fistula was considered to be cholecystopleural fistula. On the 6th day of admission, endoscopic retrograde cholangiopancreatography (ERCP) + cholecystography + Oddi sphincterotomy + laminating biliary stent was performed in the emergency department, and cholecystopleural fistula was confirmed during the operation.

**Outcomes:**

The patient recovered well after surgery, computed tomography examination on the 20th day after surgery indicated that pleural effusion was significantly reduced, and the patient was cured and discharged. The patient returned to the hospital 8 months after the ERCP operation to pull out the bile duct–covered stent. The patient did not complain of any discomfort after the postoperative follow-up for 3 years, and no recurrence of stones, empyema, and other conditions was found.

**Conclusion:**

Cholecystopleural fistula is one of the serious complications of acute cholecystitis, which is easy to misdiagnose clinically. If the gallbladder inflammation is severe, accompanied by pleural effusion, the pleural effusion is bili-like liquid, or the content of bilirubin is abnormally elevated, the existence of the disease should be considered. Once the diagnosis is clear, active surgical intervention is needed to reduce the occurrence of complications. Endoscopic therapy (ERCP) can be used as both a diagnostic method and an important minimally invasive treatment.

## 1. Introduction

Gallstone with acute cholecystitis is one of the most common diseases in the clinic. If the disease is serious, gallbladder gangrene, perforation, and sepsis may be caused. Acute gangrenous cholecystitis is a special type of cholecystitis with a high incidence. The cause of the disease may be obstruction of bile discharge caused by the incarceration of gallstones, resulting in excessive pressure in the gallbladder cavity, resulting in pressure on the gallbladder wall and ischemia and necrosis. If the treatment is not timely, it may lead to abdominal abscess, gallbladder perforation, and other severe diseases, which seriously affects the life safety of patients.^[1,2]^ Since the chest and abdominal cavity are naturally divided into 2 independent closed spaces by the diaphragm, biliary tract diseases rarely cause thoracic-related complications, especially cholecystopleural fistula, and similar cases have rarely been reported clinically.^[3,4]^ Previous literature reports mainly focused on choledopleural fistula, the causes of which were mainly related to trauma and severe surgical complications, such as chest and abdominal trauma, liver surgery, and percutaneous hepatic interventional therapy,^[5–7]^ while <10 cases of cholecystopleural fistula caused by nonsurgical reasons were reported in the literature. Here is a patient we encountered reported as follows.

## 2. Case report

### 2.1. Patient history

A 52-year-old male patient was admitted to the emergency hospital on October 30, 2019, due to repeated right middle and upper abdominal pain for 1 month.

### 2.2. Clinical findings

Physical examination showed no yellow skin sclera staining, no obvious cardiopulmonary abnormalities, a flat abdomen, right middle and upper abdominal tenderness, accompanied by rebound pain and muscle tension, presence of liver dullness boundary, slight beating pain in the liver area, positive Murphy sign, and negative mobile dullness.

### 2.3. Diagnostic workup

Abdominal color ultrasonography: gallstone, gallbladder growth, and gallbladder wall thickening of 0.9 cm. Blood routine: leukocyte: 22.16 × 10^9^/L, neutrophil percentage: 91.1%, hypersensitive C-reactive protein: 272.6 mg/L, and liver function indicated normal transaminase and bilirubin. Gallstones with acute cholecystitis were considered for admission.

### 2.4. Treatment

**Figure 1.**
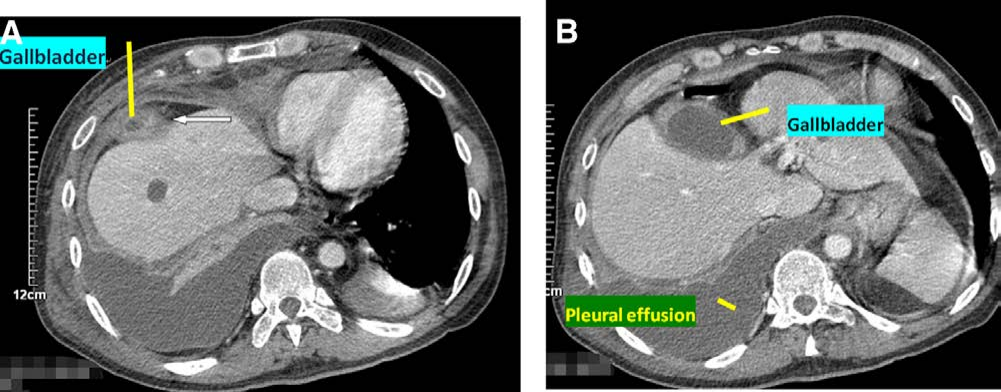
(A and B) Computed tomography showed cholecystitis and peri-inflammation with a small abscess around the base of the gallbladder, local peritonitis, a small amount of fluid in the abdominal cavity, and bilateral pleural effusion. The white arrow indicates a small abscess around the gallbladder. The yellow oblique line indicates cholecystitis with pericholecystitis.

**Figure 2.**
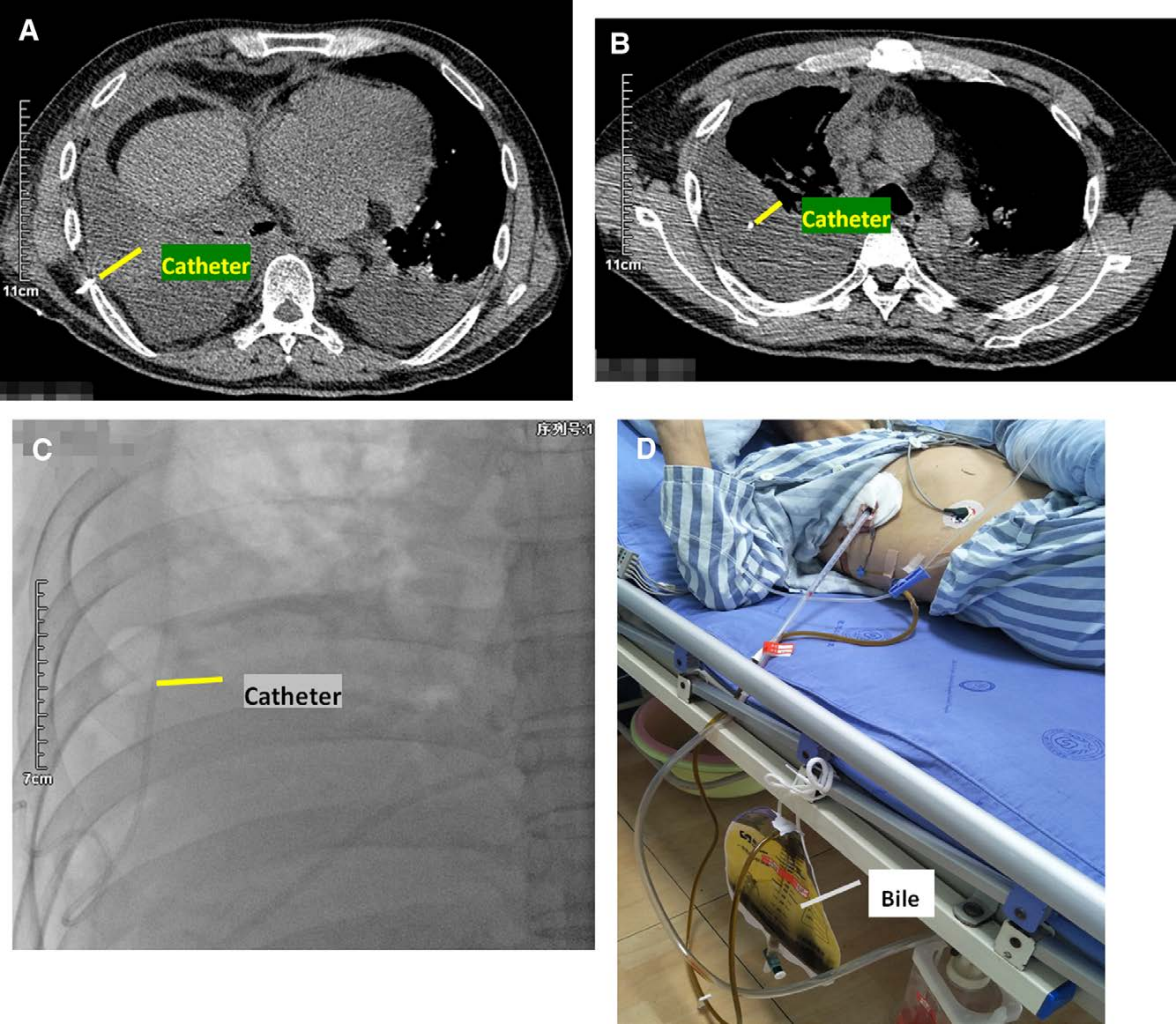
(A and B) Chest computed tomography showing a right pleural effusion and the pleural puncture catheter (as indicated by the line). (C) X-ray shows that the tip of the catheter is facing the head side, which can exclude the bile duct from entering the chest through the diaphragm. (D) A large amount of bile was drained from the catheter in the patient’s chest cavity.

After 4 days of antiinfectional treatment, the patient developed abdominal pain aggravated and then jaundice (total bilirubin: 68.0 μmol/L, direct bilirubin: 42.3 μmol/L), chills and fever (39.0 °C), chest distress, chest pain, heart fatigue and other symptoms, and heart rate of 130 times/min; emergency computed tomography (CT) indicated cholecystitis and peri-inflammation with a small abscess around the base of the gallbladder, local peritonitis, and a small amount of fluid in the abdominal cavity (Fig. 1A and 1B). On the 4th day of admission, the emergency department underwent right thoracic catheter drainage and successfully extracted 900 mL of dark yellow fluid. After surgery, bili-like fluid appeared in the thoracic drainage tube of the patient (about 2000 mL of bili-like thoracic fluid was drained out on the 1st day, and 400 mL of bili-like thoracic fluid was drained out on the 2nd day). Considering that the bili-like thoracic cavity was connected to the thoracic cavity, the patient was considered to have a very rare biliopleural fistula (cholecystopleural fistula) after iatrogenic bile fistula caused by thoracic puncture was excluded (Fig. 2A–2D).

**Figure 3.**
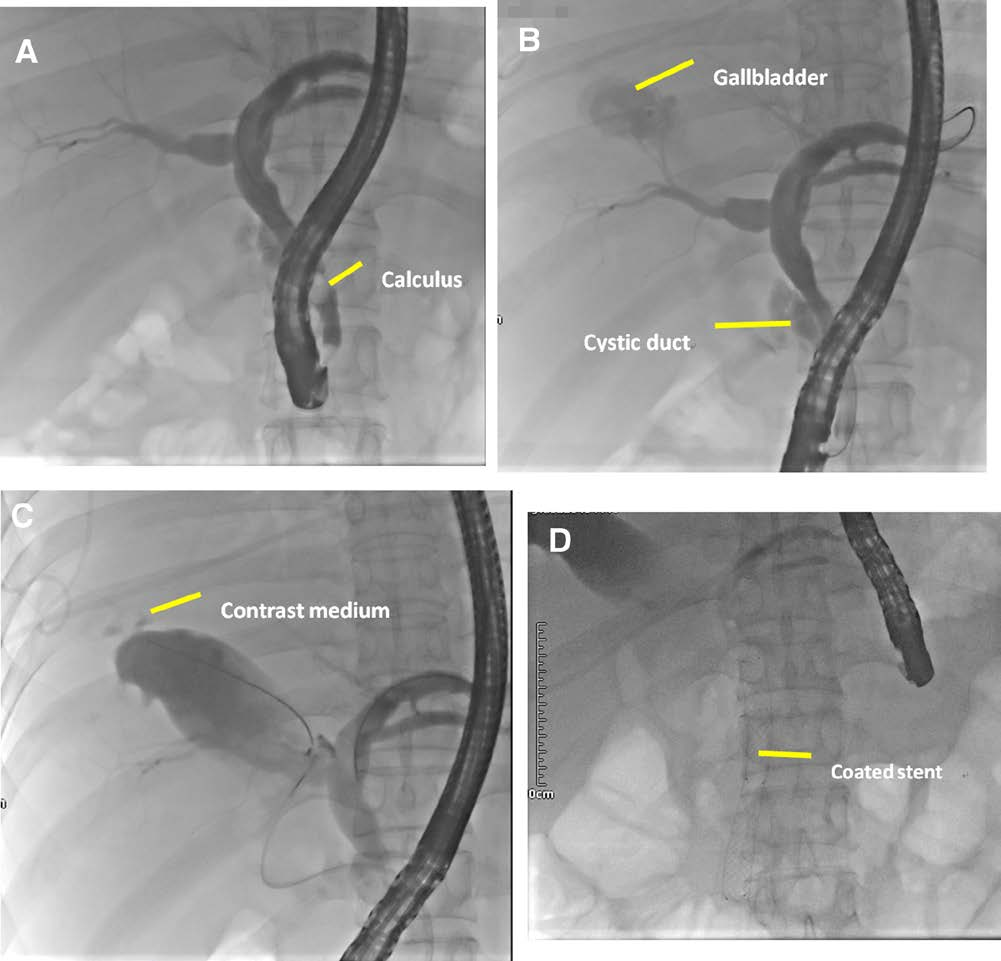
(A) The line shows choledocholithiasis, and the gallbladder was not clearly developed in the early stage of angiography. (B) After the end of the common bile duct was blocked and the angiographic pressure was increased, the gallbladder and the gallbladder duct showed obvious swelling, with the base of the gallbladder facing upward. (C) The structure of the gallbladder showed local irregularization at the base of the gallbladder and leakage of the contrast agent into the chest after injection of the arcuate knife into the gallbladder. (D) After the choledocholithiasis was removed, a coated stent was placed at the end of the choledochal duct.

**Figure 4.**
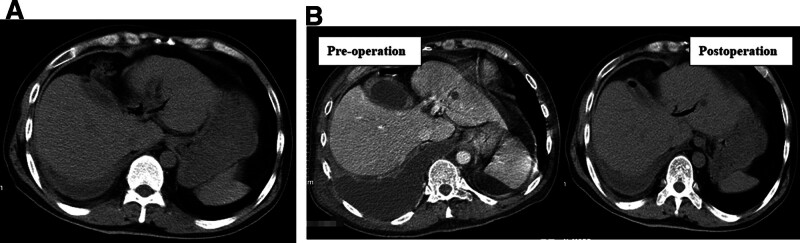
(A) Computed tomography (CT) examination 20 d after surgery indicated that most pleural effusions was absorbed, gallbladder volume was reduced, and perigallbladder inflammation was gradually absorbed. (B) Compared with preoperative and postoperative CT scans, perigallbladder inflammation and pleural effusion were significantly reduced. Due to the placement of bile duct stents in the bile duct, there was a little air accumulation in the left intrahepatic bile duct and gallbladder after surgery.

On the 6th day of admission, endoscopic retrograde cholangiopancreatography (ERCP) and cholecystography + Oddi sphincterolithotomy + coated biliary stent were performed under local anesthesia. Intraoperative conditions: intrahepatic bile duct development and mild dilatation were observed under fluoroscopy. Hepatic duct and common bile duct dilatation with a diameter of about 1.3 cm were observed. A round stone floating shadow with a diameter of about 1.1 cm was observed in the middle of the common bile duct (Fig. 3A). The cholecystic duct was developed with obvious enlargement, confluent in the common bile duct at the lower level, and the base of the gallbladder upward. The guide wire and the arcuate knife were sent into the gallbladder, and the contrast agent was injected. It was clear that there was external leakage of contrast agent at the base of the gallbladder, and the tip of the fistula of the gallbladder was toward the upper thoracic cavity (external leakage of contrast agent was observed in the gallbladder, and a large amount of bili-like liquid was observed in the thoracic cavity, suggesting a cholecystopleural cavity fistula; Fig. 3B and 3C). The calculi in the common bile duct were removed completely with a net basket. Under the guidance of the guide wire, 1 covered bile duct stent (10 × 80mm) was placed in the common bile duct. The distal end of the covered bile duct was placed in the upper segment of the hepatic duct (more than 2 cm from the left and right hepatic ducts), and the proximal end of the covered bile duct was placed in the duodenum, exceeding 2.0 cm (Fig. 3D). The postoperative recovery of the patient was satisfactory, and the fluid in the thoracic drainage tube gradually decreased. However, the reexamination of chest CT indicated that the patient was complicated with right empyema and apoptosis, and self-healing after active symptomatic treatment. The reexamination of CT 20 days after ERCP indicated that pleural effusion was significantly reduced (Fig. 4A and 4B), and the patient was cured and discharged.

### 2.5. Follow-up visit

The patient returned to the hospital 8 months after the ERCP operation and was guided by digital subtraction angiography to pull out the biliary-coated stent and clean up the stones. The patient did not complain of any discomfort for 3 consecutive years after the operation, and no stone recurrence, empyema, and other conditions were found.

## 3. Discuss

Acute cholecystitis is an acute inflammatory process of the gallbladder, and serious complications can lead to empyema, gangrene, gallbladder perforation, and sepsis.^[8,9]^ Combining the Tokyo Manual, the statements of scholars such as Neimeier,^[10–12]^ based on the duration and severity of the gallbladder perforation, it can be divided into 3 types of gallbladder perforation: chronic perforation with fistula formation (type I), subacute perforation with adhesions containing peripheral abscess (type II), and acute perforation and efflux into the cavity with systemic cholangitis (type III). In this case, the cholecystopleural fistula was between type I and type II due to gallbladder gangrene and the formation of an abscess in the surrounding tissues, which then infiltrated the diaphragm and formed a cholecystopleural fistula.

Biliary pleural fistula is the pathological communication between the biliary system and the pleural cavity, through which bile can enter the pleural cavity and form biliary pleural effusion, which is one of the serious complications of liver and bile diseases and can be divided into biliary pleural fistula and cholecystopleural fistula. This disease is commonly seen in intrahepatic bile duct inflammation, gallbladder inflammation, and diaphragmatic invasion and communication, and bile is caused by the rupture of the diaphragm into the chest. Due to the occult and rare symptoms in the clinic, especially cholecystopleural fistula,^[13,14]^ it is easy to be missed and misdiagnosed, and once it occurs, it can cause serious consequences.^[15,16]^ By reviewing the clinical characteristics of a patient with cholecystopleural fistula admitted to our hospital, this article aims to arouse the attention of clinicians to cholecystopleural fistula and improve the diagnosis rate of cholecystopleural fistula.

There are many causes of biliary pleural fistula, mainly including amebic liver abscess, biliary liver abscess, intrahepatic bile duct or common bile duct calculus, biliary ascariasis, post-radiofrequency ablation, and trauma.^[17,18]^ Literature has reported that trauma has become the main cause of biliary pleural fistula in Western developed countries,^[19]^ while obstruction caused by liver abscess and biliary calculus is more common in developing countries.^[20]^ The possible pathogenesis is a long-term intrahepatic bile duct or common bile duct stone and other bile duct history leading to repeated bile duct infection or abscess, rupture to the surface of the liver, perforating the diaphragm, and eventually forming a biliary pleural fistula.

Chronic perforation and fistula formation of the gallbladder represent an extremely rare and severe complication of gallbladder disorders in clinical practice. It can present as gallbladder-colon fistula, gallbladder-duodenal fistula, gallbladder-gastric fistula, gallbladder-skin fistula, fistula between the gallbladder and the bile duct (Mirizzi syndrome), and gallbladder pleural fistula.^[5]^ The majority of cases are induced by gallstones. Chronic perforation of the gallbladder with fistula formation allows bile to be discharged into the digestive tract through abnormal channels. After the biliary pressure decreases, clinical symptoms such as abdominal pain disappear; the condition alleviates; at the same time, gallbladder atrophy may occur; and even features such as pneumobilia may appear. Compared with chronic perforation of the gallbladder with fistula formation caused by other cases, the manifestations and prognosis of this case of gallbladder pleural fistula are different. Its manifestations are unique because the bile of this patient fails to enter the normal channels of the human body and cannot be discharged. Instead, it enters the thoracic cavity and is accompanied by a series of complications. The pathogenesis was mainly caused by stone incarceration in the neck of the gallbladder; the high tension of the gallbladder led to the change of ischemic gangrene in the gallbladder wall, and then, a local abscess formed around the gallbladder. The base of the gallbladder of the patient was oriented towards the diaphragm, and the perigallbladder abscess and diaphragm invaded and broke bile into the chest. The chest cavity is in a closed negative pressure environment, and the negative pressure is more obvious during inspiration. The patient develops secondary choledocholithiasis, biliary obstruction, and difficulty in bile excretion, and the biliary tract is under high pressure. Under the condition of negative chest pressure, bile constantly enters the chest cavity and forms biliary pleural effusion with the change of respiration.

Considering that bile is highly irritating, bile enters the chest and stimulates the pleura to produce chemical reactions. The clinical manifestations of cholecystopleural fistula are chest tightness, dyspnea, empyema formation or accompanied by chills, high fever, sepsis, etc, which are life-threatening in severe cases. If the ultrasound examination found pleural effusion, yellow-green fluid was drained by pleural puncture, and the laboratory examination of pleural effusion showed an elevated bilirubin level,^[21,22]^ then the possibility of biliary pleural fistula should be considered. Because CT and magnetic resonance cholangiopancreatography are noninvasive, they can assist in examination and make clear diagnosis, but they lack accuracy. ERCP can be used not only as an important examination but also as a means of treatment, which is widely used in clinical practice. Angiography can show the location and size of the fistula, as well as the identification of distal biliary obstruction, which is of significant help in diagnosis and treatment.^[7,23]^ In this group of patients, the fistula was clearly indicated by ERCP cholangiography, and the diagnosis could be confirmed by preoperative CT examination and clinical analysis. In this case, after ERCP cholangiography, it was found that contrast agent overflow confirmed the existence of a fistula, which, combined with preoperative imaging and clinical manifestations analysis, is of great significance for our diagnosis and treatment.

Because cholecystopleural fistula is rare and its pathogenesis is complex, it needs to be clinically distinguished from the following diseases. Biliary and bronchial fistula: the main feature of this disease is that the biliary system communicates with the bronchus; bile enters the bronchus directly; and cough, chest tightness, dyspnea, and coughing up bili-like sputum are clinical manifestations. The disease is generally associated with infection, tumor, surgery, chest, and abdominal trauma.^[24–26]^ Biliary pleural fistula: the main feature of this disease is that in the biliary system branches and diaphragm infiltration (or diaphragm perforation), the biliary system communicates with the chest cavity, and bile enters the pleural cavity, causing bili-like effusion in the chest cavity. The main manifestations of acute respiratory distress syndrome were chest tightness, dyspnea, pleural effusion, and no bilious sputum. This disease is commonly seen in the complications of percutaneous hepatic interventional surgery,^[27,28]^ such as percutaneous hepatobiliary puncture through the chest and diaphragm. After radiofrequency ablation, the needle path damaged the diaphragm, or liver abscess, biliary tract infection, and fistula formed in the diaphragm into the chest. Reactive pleural effusion: after infection of the upper abdomen, inflammatory substances enter the chest through the diaphragm and cause reactive pleural effusion. The effusion is clear in color and low in bilirubin. The puncture fluid can be identified according to its nature.

The treatment options for biliary pleural fistula include conservative treatment (thoracic drainage)^[29]^ and surgical treatment. However, considering the highly corrosive bile and obstruction at the end of the bile duct, the self-healing effect of thoracic drainage alone is poor, and serious complications such as empyema and sepsis may occur if the treatment is not timely. Therefore, surgical treatment is still the best treatment option, and the cure rate can reach 98%.^[30]^ The treatment principle of cholecystopleural fistula is “lower thinning and upper blocking,” that is, to remove biliary obstruction, repair the fistula, and resect the fistula. Traditional surgical operations require cholecystectomy, abscess removal, fistula resection, diaphragm repair, bile duct incision and exploration, and T-tube drainage. In severe cases, thoracotomy combined with thoracotomy is required. Such operations have large wounds and slow recovery, especially in unclear local structures, which are prone to damage adjacent organs and lead to more serious complications or even multiple operations. This procedure is no longer the 1st choice for the treatment of the disease. With the continuous improvement and widespread promotion of endoscopic technology, ERCP is not only a diagnostic method but also an effective means to treat cholecystopleural fistula,^[31]^ and traditional surgery has been replaced by this surgical method. In the case of this patient, an Oddi sphincter incision was performed during the operation to remove stones and relieve biliary obstruction. After implantation of a biliary-coated stent, on the one hand, the opening of the gallbladder duct could be blocked to reduce bile entering the gallbladder and then flowing into the chest cavity; on the other hand, the bile duct could be dredged to keep bile drainage smooth, so as to achieve the purpose of blocking the gallbladder duct and dredge the bile duct, which fully embodies the concept of minimally invasive surgery. Because the number of cases of this type is too small and there are only sporadic reports, there are no corresponding guidelines and expert consensus to provide reference for treatment. It is hoped that future guidelines or expert consensus can provide more treatment means to help clinicians make choices.

As for biliary drainage, according to personal experience, external nasobiliary drainage is generally not selected. Although it is more convenient to pull out the nasobiliary duct, cholecystopleural fistula treatment is a relatively long process, maintaining bile drainage for a long time, and patients are difficult to tolerate long-term nasobiliary duct treatment. Therefore, most patients are treated with stents in clinical practice. For the choice of bile duct stent, large diameter bile duct plastic stent (10 French) or coated metal stent can be selected, about the choice of the two; each has advantages and disadvantages: (1) plastic stent: advantages: cheap; disadvantages: the stent is easy to block and shift, short-time replacement (generally 3–6 months), and if the patient’s cholecystopleural fistula treatment effect is long, plastic stent replacement will bring more economic burden and trauma to the patient. (2) Coated metal bracket: disadvantages: the price is expensive and may shift. Advantages: the coated metal stent has a large aperture, the drainage effect is exact, and the patency time can reach >1 year and generally does not need to be replaced. Another purpose of choosing a coated stent is to consider that the gallbladder duct of this patient is lower than the conjoint common bile duct, and the support of the coated stent can block the gallbladder duct opening, reduce bile entering the gallbladder, and fully allow the cholecystopleural fistula to heal itself.

## 4. Conclusion

Cholecystopleural fistula is a very rare and serious complication of acute cholecystitis, and it is easy to be misdiagnosed clinically. If the gallbladder inflammation is severe, accompanied by pleural effusion, and the pleural effusion is bili-like liquid or the content of bilirubin is abnormally elevated, the existence of the disease should be considered. Once the diagnosis is clear, active surgical intervention is needed to reduce the occurrence of complications. Combined with our experience, endoscopic therapy (ERCP) can be used as both a diagnostic method and an important minimally invasive treatment option, which can be actively carried out and applied in experienced endoscopic therapy centers.

## Author contributions

**Conceptualization:** Yong Yang

**Visualization:** Qian Chen

**Writing — review & editing:** Qian Chen

**Data curation:** Yi Hu

**Supervision:** Liangsong Zhao

**Validation:** Pengcheng Cai

**Methodology:** Suqi Guo
